# Contraceptive Methods Accessed in Volta Region, Ghana, 2009–2014

**DOI:** 10.1155/2017/7257042

**Published:** 2017-09-07

**Authors:** Himiede W. Wilson, Donne K. Ameme, Olayinka Stephen Ilesanmi

**Affiliations:** ^1^National Public Health Institute of Liberia, Monrovia, Liberia; ^2^Ghana Field Epidemiology and Laboratory Training Program, Department of Epidemiology and Disease Control, School of Public Health, College of Health Sciences, University of Ghana, Accra, Ghana; ^3^Liberia Field Epidemiology Training Program, Monrovia, Liberia

## Abstract

**Introduction:**

In 2016, Volta Region was one of the two regions in Ghana that recorded a high prevalence of teenage pregnancy, accounting for 15.5% of all adolescent pregnancies in the country. This study aimed to determine the prevalence of contraceptive methods accessed by person, place, and time in Volta Region, Ghana, 2009–2014.

**Method:**

We carried out a secondary analysis of contraceptive use data derived from the District Health Information Management System (DHIMS) 2 for Volta Region, between 2009 and 2014. We calculated proportions and described trends.

**Results:**

Over the five-year period, there were 673,409 (75.0%) acceptors of family planning out of a total 897, 645 males and females of reproductive age. The proportion of family planning acceptors increased gradually from 18% in 2009 to 23% in 2014. Contraceptive methods were most commonly accessed by male and female between 20 and 29 years. The most common methods of contraceptives accessed were injectables among females accounting for about 70% and condoms accounting for over 90% among males.

**Conclusion:**

All the districts in Volta Region did not access contraceptives adequately. The Volta Regional Health Directorate should encourage and support research to ascertain factors influencing uptake of contraceptive methods in all the districts.

## 1. Introduction

Accessing contraceptive methods is a major component of family planning. It employs the uptake of one of the various methods, devices, drugs, sexual practices, or surgical procedures to prevent conception or impregnation [1]. These various methods can be grouped into long-term and short-term methods. Long-term method include intrauterine devices, implants, and sterilization which is to limit child bearing and short-term such as pills, condoms, spermicides, and injectables [2]. Other modern and traditional methods are used by women who want to delay but not lose having a child. The use of contraceptive is a crucial aspect of health services and it benefits health and welfare of women, men, children, families their communities, and the country at large [3].

Contraceptive use has risen in many parts of the world mainly in Asia and Latin America but continuously low in sub-Sahara Africa. Globally the uses of modern contraceptive have risen slightly from 54% in 1990 to 57.4% in 2014. Regionally, in sub-Sahara Africa, the proportion of women aged 15–49 years reporting use of a modern contraceptive method has increased little between 2008 and 2014 from 23.6% to 27.6%. The use of contraception by men makes up a relatively small subset of the prevalence rate stated above. Modern contraceptive methods for men are limited to male condoms and sterilization (vasectomy) [4]. The increase rate of contraception around the world has afforded couples the ability to choose the number and spacing of their children. Also among youths it helps prevent unplanned pregnancies, unsafe abortions, sexually transmitted infections (STIs), and maternal deaths [5].

According to worldwide estimates, 600,000 women die each year of pregnancy related causes and 75,000 die as a result of unsafe abortions. About 200,000 of these maternal deaths are attributable to the failure or lack of contraceptive services [6]. Contraceptive is one of the proximate determinant factors of fertility [7]. The United Nation Millennium Development goals (MDGs 1 to 7) are connected to family planning. The emphasis is mainly placed on MDG 5, whose aim is to improve maternal health and reduce maternal mortality by three-quarters between 1990 and 2015. The promotion of modern contraceptive methods among women in sub-Sahara Africa is an important intervention gear toward achieving the target. The expansion of assessment of family planning has been an important aim of health and development for almost fifty years [8]. There is a need to know the prevalence and trend of contraceptive use to guide intervention to promote its use. This study aimed to determine the prevalence of contraceptive accessed in the Volta Region of Ghana by person, place, and time.

## 2. Methods

### 2.1. Study Area

The Volta Region is one of the ten regions in Ghana. It is located along the southern half of the eastern border of Ghana that is shared with the Republic of Togo. In the west, it shares boundaries with Greater Accra and Eastern and Brong Ahafo regions, in the north with the Northern Region, and in the south with the Gulf of Guinea. The region occupies an area of about 20,570 square kilometres or 8.6 per cent of the total land area of Ghana. It has a population of 2, 33799 and 25 districts. It has a fertility rate of 3.8. The region has 21 public hospitals, 8 private hospitals, 153 health facilities, and 265 chps compounds. It has a population of 427,556 males and 470,089 females aged 15–45 years.

### 2.2. Study Design

This study used a cross-sectional retrospective design. It involved secondary data collection from records of males and females sexually active aged 15–45 years accessing a form of contraceptive method at a health facility between 2009 and 2014.

### 2.3. Data Collection

Secondary data was extracted and reviewed to determine the prevalence of contraceptive method accessed in Volta Region. The Regional Health Directorate of the Volta Region collects aggregated data on contraceptive methods accessed through the monthly family planning return form B, from the reproductive and child health unit at all health facilities in the region unto the District Health Information Management System (DHIMS) 2. Three-year data was downloaded to an Excel spreadsheet from the DHIMS 2 covering the period 2012–2014, while data of periods 2009 and 2011 was manually extracted from the yearly regional reproductive and child health reports, respectively. The data was entered and cleaned in Microsoft Excel.

### 2.4. Data Analysis

The data was analyzed in Microsoft Excel. The population denominator for each contraceptive method accessed by districts was total acceptors for each contraceptive method. The population denominator for contraceptive method accessed by region was further broken down as follows: condom use which include male and female, population denominator was the total acceptors of condoms. There are other methods such as Foaming pills, Jadelle, Implanon, Sino-implants, LAM, IUD, Norigynon, Depo, Bilateral Tubal Ligation, Minipills, Emergency Contraceptive, Micro-N, Female Sterilization, Postinor 2, Micro-G, Ovrette, Lo-Fem, Levonorgestrel Intrauterine System and Micro-lut. Population denominator was calculated by subtracting male condoms and vasectomy method from the total acceptors of contraceptive method for the particular year. These methods were for female population. The population denominator for natural method was the total population of acceptors for the particular year.

The data was analyzed and presented descriptively in percentages, graphs, distribution tables and rate to show trend (2009–2014), coverage (2012–2014), rate of new acceptors (2012–2014), new and continuing acceptors (2009–2014), contraceptive methods accessed by districts & region (2009–2014), contraceptive method accessed by months 2012–2014 and age categories of acceptors (2012–2014).

## 3. Results

**Figure 1:**
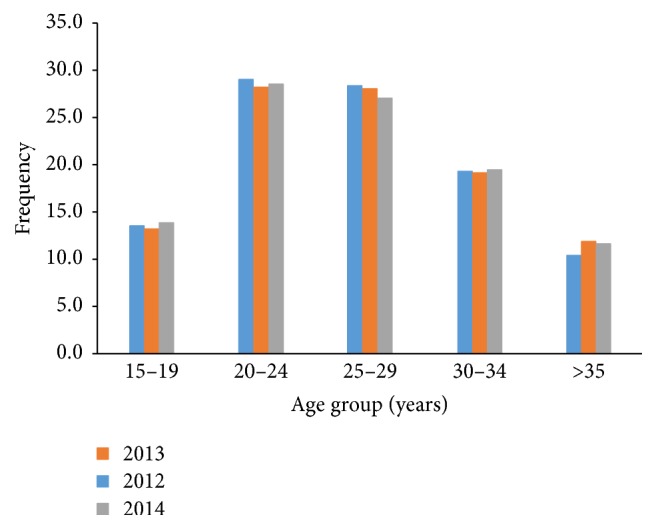
Age distribution of contraceptives acceptors in Volta Region, Ghana, 2012–2014.

Figure 1 shows the age distribution of contraceptives acceptors. Age category 20–24 years were the most acceptors over the three years in review, while the age group > 35 were the lowest acceptors.

**Figure 2:**
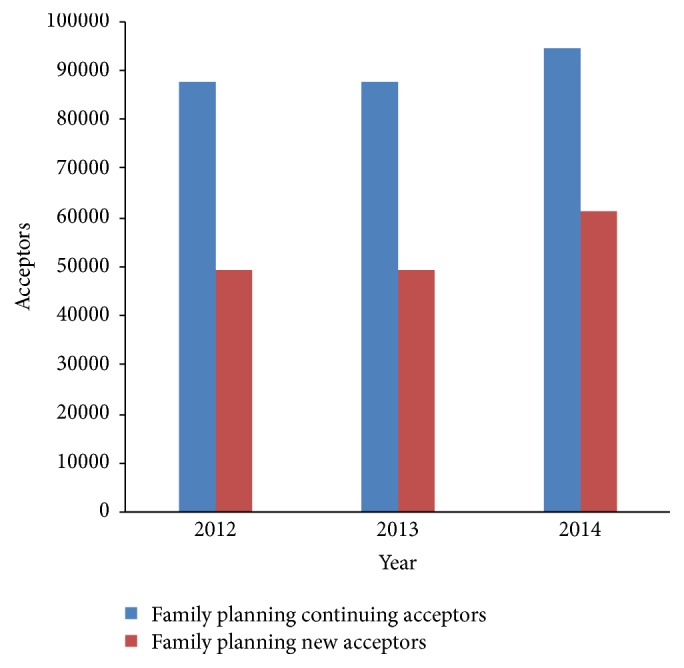
New and continuing contraceptives acceptors in Volta Region, 2012–2014.

The number of new and continuing acceptors of contraceptive method accessed at all health facilities in Volta Region increased over the years. The number of continuing acceptors was higher compared to new acceptors. New and continuing acceptors are shown in Figure 2.

Table 1 shows the rate of new contraceptives acceptors by districts in Volta Region, 2012–2014. The rate was high in Keta and Biakoye Districts. Districts that recorded the lowest rate were Hohoe, Krachi East, and Akasti South.

**Figure 3:**
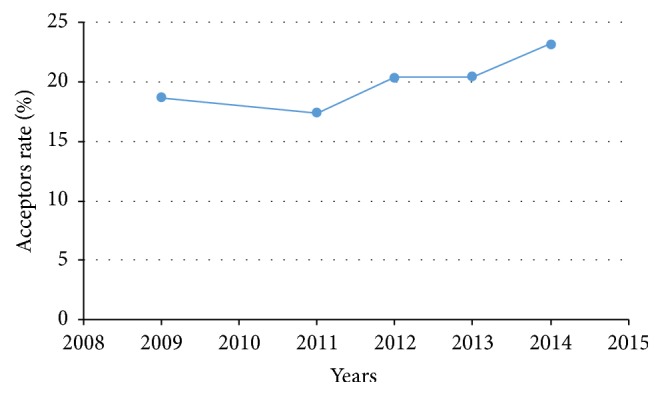
Trend of acceptors of contraceptive methods in Volta Region, 2009–2014.

The trend of contraceptive method accessed by male and female aged 15–45 years in Volta region covering period 2009–2014 is shown in Figure 3. The rate dropped from 19% in 2009 to 17% in 2011. There was a gradual peak in the number of acceptors from 2011 (20%) to 2014 (23%).

The total number of contraceptives acceptors by month among men and women 15–45 years in Volta region was highest in January of each year. All other months recorded relatively the same figures.  Table 2 shows the distribution of contraceptives acceptors by months in Volta Region, 2012–2014.

Table 3 shows the total contraceptive method accessed by male and female aged 15–45 years in districts of the Volta region. Over the five-year period, the uptake varies by districts. In 2009, Ho recorded the highest number of acceptors accounting for 13.3%. Also, in 2011, Ho was seen to have recorded the highest number of acceptors. In 2012, Keta district recorded the highest acceptors accounting for 12%. In 2013, Ketu South recorded the highest number of acceptors accounting for 12% and in 2014 the highest number of acceptors was recorded in Ho district accounting for 8%.

Among all the districts, contraceptive coverage was highest in Krachi West (61.1%) in 2012, Jasikan (52.7%) in 2013, and North Dayi (85.7%) in 2014. The contraceptive coverage for each district from 2012 to 2014 was as shown in Table 4

Among all the contraceptive methods accessed by male and female aged 15–45 years in the 25 districts, male condoms and Depo-Provera (injectable) were the two methods commonly accessed. Other contraceptive methods accessed are shown in Table 5.

## 4. Discussion

Contraceptive data was collected and analyzed on variables such as age, contraceptive methods, districts of acceptors, and new and continuing acceptors. The use of any form of contraception has increased over the years from 18% in 2009 to 23% in 2014. Contraceptives use over the last six years has increased from 24% to 27% [2]. The percentage of acceptors was high in Ho, Keta, and Ketu South Districts between 2009 and 2014 compared to Akatsi North and South Districts that remain low accounting for 2.47%  and 1.71%, respectively, as these districts are urban. Living in urban area has been linked to higher knowledge of contraceptives [9]. This is similar to a study stating that knowledge of contraceptive method and knowledge of access to services were significantly associated with the uptake of contraceptive method [10].

Age had a link with contraceptive methods accessed in Volta Region over the five years. The age category mostly accessing contraceptive method was 20–24 years. And ages 15–19 and >35 years were the lowest registrants accessing contraceptive methods. It has been showed that age categories of 15–19 and 40–44 years were the highest age categories not accessing contraceptive method [11]. Despite the importance of contraceptives, its use in older and perimenopausal women is lower than younger women [12]. The commonly used contraceptive methods were Depo-Provera (injectable) and male condom in Volta Region. Male condom with 94% was proven to account for the highest method accessed by males. On the contrary, pills method was the highest contraceptive method accessed by females [11].

## 5. Conclusion

Practices of contraceptive method are crucial to family planning, as it helps couples and teenagers plan and have children by choice. Also practice of contraception prevents acceptors from unintended pregnancies and unsafe abortion. This will benefit families, communities, and the country at large. The access to contraceptive methods has increased gradually over the years.

The Ghana Health Services should revise the family planning reporting form of the District Health Information Management System (DHIMS) 2 to capture other variables such as occupation, number of children, and education level to inform policy maker as to target population at high risk. The Volta Regional Health Directorate should encourage and support research to establish factors influencing uptake of contraceptive methods in urban and rural districts.

## Figures and Tables

**Table 1:** Rate of new contraceptives acceptors by districts Volta Region, 2012–2014.

Districts	2012	2013	2014
Adaklu	9.8	12.9	22.1
Afadjato South	6.2	5	6.3
Agortime Ziope	7.4	13.9	27.6
Akatsi North	0	12.9	15.6
Akatsi South	2.9	3.8	2.8
Biakoye	15.9	**23**	**39.6**
Central Tongu	3.3	4.5	15.9
Ho	7.4	8.9	11
Ho West	12.2	12.8	15.2
Hohoe	2.5	4.2	4.6
Jasikan	13.4	13	13.1
Kadjebi	7.5	6.3	7.3
Keta	**21.7**	17.5	9.4
Ketu North	6.3	7.8	8.8
Ketu South	4.1	4.6	4.2
Kpando	11.2	4.4	6.1
Krachi East	3.1	3.7	4
Krachi Nchumuru	9.3	6.8	8.7
Krachi West	16.9	5.8	9
Nkwanta North	12.3	15.1	18.8
Nkwanta South	18.7	13.4	14.2
North Dayi	10.9	14.2	34
North Tongu	8.1	4.3	3.2
South Dayi	8.8	7.2	11.9
South Tongu	14.6	15.1	15.2

**Table 2:** Distribution of contraceptives acceptors by months in Volta Region, 2012–2014.

Months	2012		2013		2014	
	*n*	%	*n*	%	*n*	%
January	14,789	**10.79**	14,236	**10.39**	16,176	**10.37**
February	13,819	10.08	11,767	8.58	13,338	8.55
March	13,110	9.56	10,618	7.75	13,608	8.72
April	11,383	8.30	11,758	8.58	11,987	7.68
May	10,215	7.45	11,224	8.19	13,929	8.93
June	11,081	8.08	10,005	7.30	12,991	8.33
July	11,802	8.61	11,000	8.03	12,464	7.99
August	10,989	8.02	10,976	8.01	13,165	8.44
September	10,092	7.36	10,409	7.59	12,481	8.00
October	11,056	8.07	11,878	8.67	12,440	7.97
November	9,350	6.82	11,455	8.36	11,841	7.59
December	9,382	6.84	12,106	8.83	11,577	7.42

Total	**137,068**	**100**	**137,432**	**100**	**155,997**	**100**

**Table 3:** Distribution of contraceptive methods accessed by districts in Volta Region, 2012–2014.

Districts	2009 %	2011 %	2012 %	2013 %	2014 %
Adaklu	9.77	5.24	1.99	2.57	3.41
Afadjato south	0.00	0.00	3.14	3.97	4.17
Agortime Ziope	0.00	0.00	1.79	5.98	3.65
Akatsi North	0.00	0.00	0.00	3.34	2.47
Akatsi South	0.00	0.00	1.51	1.11	1.71
Biakoye	0.40	1.89	3.71	2.66	7.47
Central Tongu	0.00	0.00	1.76	6.76	2.57
Ho	13.27	13.14	9.39	5.17	8.24
Ho West	0.00	0.00	5.29	2.92	4.79
Hohoe	8.42	9.39	4.35	2.91	5.02
Jasikan	3.87	2.33	5.13	2.61	5.57
Kadjebi	3.13	3.29	2.85	1.68	2.83
Keta	9.92	11.64	12.24	3.52	4.94
Ketu North	1.63	2.04	2.37	3.87	3.85
Ketu South	0.00	0.00	3.55	12.12	3.07
Kpando	12.17	4.54	3.94	3.05	1.77
Krachi East	3.14	2.74	2.16	5.87	1.67
Krachi Nchumuru	0.00	0.00	3.29	5.70	3.39
Krachi West	3.86	9.24	6.27	4.22	2.94
Nkwanta North	3.69	6.71	3.43	7.12	5.98
Nkwanta South	0.00	0.00	9.10	0.90	5.61
North Dayi	0.00	0.00	2.26	4.90	5.81
North Tongu	4.70	3.30	2.70	2.42	0.66
South Dayi	2.57	2.27	3.14	2.24	3.76
South Tongu	3.66	5.81	4.63	2.41	4.66
Nkwanta	8.32	7.30	0.00	0.00	0.00
Akatsi	3.12	4.93	0.00	0.00	0.00
ketu	4.35	4.21	0.00	0.00	0.00

**Table 4:** Distribution of contraceptive methods accessed by districts in Volta Region, 2012–2014.

Districts	2012 %	2013 %	2014 %
Adaklu	32.2	40.9	67
Afadjato South	27	23.9	25.8
Agortime Ziope	31.1	41.2	62.3
Akatsi North	—	39.2	44
Akatsi South	8.3	13.1	10.6
Biakoye	30.5	39.5	66.8
Central Tongu	14.4	7.7	25.5
Ho	28.4	21.2	27.4
Ho West	30.7	24.1	29.8
Hohoe	11.8	17.5	17.7
Jasikan	46.9	**52.7**	55.4
Kadjebi	26	27.3	28.1
Keta	44.8	43.7	19.7
Ketu North	12.8	20.6	22.7
Ketu South	12	11.6	11.3
Kpando	38.6	16.2	19.4
Krachi East	10	11.9	8.4
Krachi Nchurumu	26.7	23.2	27.4
Krachi West	**61.1**	28	35
Nkwanta North	28.7	42.6	51.1
Nkwanta South	41.8	30.5	29.9
North Dayi	31.8	36.8	**85.7**
North Tongu	17.5	6.8	4.3
South Dayi	36.4	38	47.4
SouthTongu	28.5	36.1	31.2

**Table 5:** Types of contraceptive method assess in Volta Region, 2009–2014.

Methods	2009 %	2011 %	2012 %	2013 %	2014 %
Male Condom	**98.6**	**99.2**	**96.45**	**94.51**	**94.78**
Female Condom	1.31	0.76	0.35	5.49	5.23
Vagina Foaming Tablet	0.11	0	0.32	0.09	0.08
Combined Pills	18.8	17.48	14.84	6.53	8.12
Progesterone only Pills	1.53	2.1	0.90	4.61	4.21
Intrauterine Contraceptive Device	0.54	0.7	0.31	0.17	0.26
Injectable contraceptives	74.1	70.09	67.48	67.70	61.59
Bilateral Tubal Ligation	0.51	0.68	0.34	0.19	0.21
Vasectomy	0.003	0	0.05	0.08	0.03
Implants	1.68	2.93	3.36	3.98	6.51
Natural	0.14	0.1	0.07	0.96	1.24
Lactational Amenorrhea	1.92	5.33	11.05	14.69	17.41
LNG-IUS	0	0	0.09	0.03	0.02
Emergency Contraceptive	0	0	0.68	0.43	0.07
